# The role of medical humanities in the formation of physicians who work with patients at the end-of-life

**DOI:** 10.1007/s40592-025-00255-0

**Published:** 2025-06-12

**Authors:** Xavier Symons, John Rhee

**Affiliations:** 1 Australian Catholic University, Brisbane, Australia; 2 Harvard University, Cambridge, USA; 3 Center for NeuroOncology, Department of Medical Oncology, Dana-Faber Cancer Institute, Harvard Medical School, Boston, 02215 USA; 4 Division of Adult Palliative Care, Department of Supportive Oncology, Dana-Faber Cancer Institute, Harvard Medical School, Boston, 02215 USA

**Keywords:** Death, End-of-life, Euthanasia, Brain death, Professionalism, Narrative, Medical humanities

## Abstract

The medical humanities developed in the second half of the 20th century in response to challenges of mechanisation and dehumanisation in medicine. Humanities disciplines like literature and art and social sciences like anthropology and sociology served to ameliorate reductionistic tendencies in medicine and ensured that medical trainees developed a deeper understanding of patient experiences and perspectives. The medical humanities bequeathed to medicine a capacity for self-critique and highlighted ways in which medicine could provide more humane care to patients. One challenge, however, that remains inadequately addressed is the task of providing a solid humanistic formation to physician trainees who are preparing to care for patients at the end-of-life. This essay thus considers the role of medical humanities in the formation of physician trainees who will care for dying patients. We seek to identify specific ways in which we improve the humanistic formation of doctors so as to prepare them to care for dying patients. We close with recommendations for how to adapt medical education and training so as to include a more robust immersion in medical humanities content focused on death and dying.

## Introduction

The medical humanities developed in the second half of the 20th century in response to challenges of mechanisation and dehumanisation in medicine (Fox 1985). Humanities disciplines like literature and art, in addition to social sciences like anthropology and sociology, proved to be a fruitful source of insight into the experience of human embodiment, the drama of illness, and the variety of ways in which human beings experience ill-health and, ultimately, death. The medical humanities thus served to ameliorate reductionistic tendencies in medicine and were helpful in ensuring that medical trainees developed a deeper understanding of patient experiences and how to provide whole-person care. More recently, authors have explored how ‘critical’ approaches to the medical humanities, including but not limited to insights from critical theory, can enrich our understanding of the relationship between humanities disciplines and medicine and help us to see how medicine is a socially embedded practice that is inevitably entangled with the cultural and politic dynamics of societies (Viney et al. 2015). One example of this critical turn in medical humanities is Alan Bleakley’s work on trust in medicine, which argues that medical practice needs to shift from being defined by managerialism, hierarchy, and patriarchy toward a more democratic, feminist, and collaborative model (Bleakley 2019).

One challenge, however, that remains inadequately addressed in medicine, is the task of providing a solid humanistic formation to physician trainees who are preparing to care for patients at the end-of-life. Research indicates that physician trainees often feel ill-equipped to care for patients who are dying. Medical curricula provide limited input on how to support dying patients and their families. This essay thus considers the role of medical humanities in the formation of physician trainees who will care for dying patients. We begin with a reflection on the importance of medical humanities in general. We then seek to identify specific ways in which we improve the humanistic formation of doctors to prepare them to care for dying patients, and close with recommendations for how to adapt medical education and training so as to include a more robust immersion in medical humanities content focused on death and dying.

## Why the medical humanities is especially important in physician formation today

In the late 19th and 20th centuries, there was a strong movement in medicine towards a biological model of health, which Eric Cassel labelled ‘biomedicine’, due to the rise of germ theory and advances in pathology (Cassel 1982). This movement further codified itself into medical education through the Flexner Report of 1910 that standardized medical education around a scientific, laboratory-based approach, emphasizing diagnosis and treatment of disease around a purely biological approach.

Though the biomedicine approach brought with it many advances, including antibiotics, which led to one of the greatest drops in mortality in the history of medicine, it also came with a frameshift in the physician away from a holistic view of the person to a narrow focus on the biological disease process. This approach led medicine down an increasingly reductionist path of understanding disease through breaking down the body into smaller and smaller parts: organs, tissues, cells, and cell signaling pathways. Furthermore, with advances in technology, the physician moved away from the subjective experiences of the patient as the centerpoint for treatment, to laboratory and imaging tests as the gold standard in which to diagnose and treat patients. Lastly, the influence of Cartesian dualism on medicine also led to a separation of body from the mind, leaving little room for existential questions that arise in an illness process, and especially at the end-of-life, and leaving aside the understanding of suffering as a deeply personal experience that fractures the person as a whole. This biomedicine view and approach to disease has had its consequences in deemphasizing the lived experience of illness for patients.

The biomedicine model continues to be the dominant path of medical formation in our current training model. A brief look at any US medical school curricula, for example, reflects the emphasis placed on the basic sciences in the preclinical phase and understanding disease at a biological level rather than broader humanistic perspectives, and standardized exams are limited to biomedical knowledge. Diagnosis and treatments are based on a gold standard of evidence-based guidelines which are based on clinical trials and measurable endpoints rather than patients’ individual lived experiences. In residency training, biomarkers, imaging, and labs are prioritized in the diagnosis over the subjective experience of the patient’s illness. Medical education continues to push sub-specialized care, based on its reimbursement structure and prestige, and the focus on specialist training reinforces a biological approach to specific organ systems or diseases rather than whole-person care. And the “idealized” physician today is not the expert clinician and diagnostician but rather the physician-scientist who spends a significant portion of his/her time in research, also based on a biological model.

There has been increasing interest in how to train humanistic physicians in recent decades, as reflected through organizations like the Gold Humanism Honors Society, as well as a move towards trying to diversify the medical curriculum to include topics on social determinants of health, health disparities, ethics, among others. However, the approach has not been uniform, partly due to varying priorities in a very tightly regulated medical curriculum but also due to the fact that there is disagreement in what should be included in this “soft skills” curriculum. However, what is agreed upon is that a higher emphasis on the humanistic physician is crucial, due to a widening recognition of the fact that physicians need to be skilled in the diagnosis and treatment of patients, but that the person is not only his/her disease. Rather, the subject is the patient who carries the disease and is living through all the implications of suffering through an illness that affects all aspects of who he/she is, including one’s own understanding of self and identity.

Here is where the medical humanities, which address these deeper philosophical questions, either directly through philosophy, theology, and ethics, or indirectly through arts, literature, and narrative, is core to the formation of a medical trainee. The purpose of education is not only skills-based training, but also the moral formation of the learner, broadening his or her mind to see not only the event in front of him, but also the broader context in which that event is taking place. One cannot divorce the person’s experience of illness and the illness itself. This becomes even more vital as a patient approaches the end-of-life, where the coalescing of all the events of one’s life becomes ever more urgent but also clearer, as though pieces of one’s life start to form a deeper understanding of the whole. In such a crucial state of synthesis, the final chapters of a patient’s story, the physician’s role is crucial, and the medical humanities provides the clinician with the tools to navigate, process, and reflect on the patient’s narrative within which he or she is entering.

### Challenges surrounding medical training in end of life care

Medical students encounter death and dying in a variety of ways in their training. Doctors must have conversations with patients about death and dying including breaking news to patients that they are dying or providing advice about the end-of-life– how to manage fear, how to help loved ones, what medical care to receive, and what patients would like to happen after they die. Personal factors may also condition the way in which physician trainees encounter dying and death. Physician trainees may or may not have experienced death in their own lives, and depending on the novelty of death and dying, caring for patients at the end-of-life may be easier or more difficult. Regardless, physician trainees must, in their new professional role, learn how to negotiate their personal emotions with the requirements of being a physician, which necessitates some degree of detachment and objectivity.

Medical education does not train physicians well to deal with death and dying (Sullivan et al. 2003), and the literature shows that physicians feel both ill-equipped with how to care for patients at the end-of-life and their training in end-of-life care (Brown 2019). In the curricula of most medical schools, topics on death and dying and end-of-life care only comprise a small proportion of classroom content, and most residents do not receive thorough training in end-of-life care (Sutherland 2019). The education that physician trainees do receive would appear to occur at least in part through an “informal curriculum” of observing how other more experienced trainees or physicians interact with patients who are dying and also their families (Baker et al. 2011).

The solution is not straightforward as the source of the problem runs deep within medicine. Medicine as a discipline has tended to take a very practical approach to death (partly out of necessity) but this has led to the philosophical and existential dimensions of death being set to one side in favour of streamlining end-of-life medical practices and processes. For example, the definition of death in the context of organ donation does not broach broader metaphysical questions about whether death is an event or a process and what the defining features are of a living human being. Instead, the criteria are focused on how doctors may declare death for the purposes of vital organ donation (“A Definition of Irreversible Coma: Report of the Ad Hoc Committee of the Harvard Medical School to Examine the Definition of Brain Death,” 1968). With advances in technology, such as the mechanical ventilator, the physician is now faced with the deeper realities of defining what death even is and when it occurs. Doctors may also be ambivalent about discussing spiritual and existential questions with dying patients– a task which could be viewed as being the purview of hospital chaplains. Given medicine and medical education’s limited engagement with metaphysical and existential questions, such as the spiritual dimensions of death and dying, it faces challenges in holistically forming its trainees in working with patients and families who experience the full human drama of death and dying.

This is a major problem for multiple reasons. First, medical trainees don’t know how to care well for those at the end-of-life, and yet patients expect their clinicians to know how to help guide them through the end-of-life. Second, dying can be traumatic and medical trainees don’t have the space to cope and/or process their experiences, leading to burnout and lack of empathy. Third, dying raises ethical questions, including issues of euthanasia and physician assisted suicide and brain death and medical trainees are ill-equipped to grapple with these questions because there is no good grounding in what death is and what constitutes a good death. It is so important that trainees receive comprehensive end-of-life care training, then, and yet it is largely absent. Palliative care training is one exception to the rule, but even there, only a minority of medical students get clinical or course exposure to palliative care (Van Aalst-Cohen et al. 2008). A recent survey of students and staff in Canadian palliative medicine programs found that 84% of trainees and staff physicians agreed or strongly agreed that their palliative medicine program would benefit from more humanities (Delbani et al. 2024).

## How the medical humanities can prepare physicians to accompany patients at the end-of-life

The medical humanities are one means to address medical trainees’ ‘death literacy’ problem. Humanities, perhaps most importantly, as well as art, allow for a medium through which one can enter into the perspective of another, and thereby, having a less threatening way in which one can broaden one’s perspective and also process one’s own experiences (Chisolm et al. 2021) (Gaufberg et al. 2023) (Hafferty et al. 2015) (Katz and Khoshbin 2014). The humanities also touch on some of the most important moral questions, all of which are intimately tied to the practice of medicine. At the highest level of generality, the humanities explore what it means to be human. This has implications for the goals of medicine, if we take medicine to be in some meaningful way focused on promoting human wellbeing. More specifically, the humanities can help us to reflect on the nature of the good life, and, by extension, the nature of a good death. This last question has eminently practical implications for the way in which we approach caring for patients nearing the end-of-life.

In what follows, we highlight a few examples of how the medical humanities can perform the role of helping physicians to reflect on patient experiences of dying and death and also how humanistic texts can help physicians think through the question of a good death.

One important function of the medical humanities is that it allows physicians to enter more fully into the perspective of patients on death and dying. One recent, popular literary example of this is Paul Kalanithi’s memoir *When Breath Becomes Air* (Kalanithi et al. 2016). Kalanithi was a neurosurgeon who was diagnosed with stage IV lung cancer at the age of 36. The book chronicles his journey from being a dedicated doctor to becoming a patient facing his own mortality. It explores deep themes of medicine, meaning, identity, and what it means to live a meaningful life in the face of death. The memoir was published posthumously and includes a moving epilogue by his wife, Lucy Kalanithi.

A very powerful feature of this book is that it is written from the perspective of a doctor. This makes the book particularly impactful for physicians who might otherwise think of death as something personally distant even though they may deal with dying patients professionally on a regular basis. By way of example, in the opening chapter of the book Kalanithi writes of finding himself in a hospital examination room where he had on countless occasions delivered grim prognoses to patients– only that in this case it was him who was in the position of a vulnerable patient having just received his lung cancer diagnosis. Reading about this surreal experience is tough but may also help reduce the distance that physicians might put between themselves and their daily encounters with mortality. It is not that one wants to develop an excessive preoccupation with death, but it may assist physicians in avoiding developing an unhelpful aloofness from their patients who are very ill.

The medical humanities also help physicians to grapple with the question of ‘what is a good death?’. Atul Gawande’s *Being Mortal: Medicine and What Matters in the End* is an important example of this (Gawande 2014). Gawande’s book focuses on aging, end-of-life care, and how modern medicine often prioritizes prolonging life over ensuring quality of life. Gawande, a surgeon and writer, explores how the medical system treats aging and terminal illness, often focusing on interventions that may not align with a patient’s true wishes. Through personal stories, patient experiences, and historical context, he argues for a shift toward a more compassionate approach—one that prioritizes dignity, autonomy, and meaningful living, even in the face of decline. The book encourages open conversations about death and how we can better support people in their final years.

Gawande also explores a tradition of death and dying that our modern culture has lost, what we might call the *Ars Moriendi*. He notes the prevailing separation of the elderly and the dying from the rest of society and laments the fact that we do not visit cemeteries anymore. The ultimate message of his book is one that has direct relevance to end-of-life care, namely, that we ought, as a rule, to prioritise wellbeing over doggedly pursuing survival. Gawande provides a sociologically-informed and humane account of what it means to grapple with mortality and how to help people to live their final days in peace and with happiness rather than attempting to rail against the inevitable.

This latter concern message is also explored in works of fiction like Tolstoy’s *The Death of Ivan Ilyich*, which offers a strongly moral account of what it means to die well. Tolstoy’s novella depicts the existential crisis of a man confronting his own mortality (Tolstoy 1973). It follows Ivan Ilyich, a high-ranking Russian judge who has lived a life focused on social status, career success, and material comforts. When he falls terminally ill, he begins to reflect on the emptiness of his existence, realizing that he has prioritized superficial achievements over genuine human connection. As he suffers physically and emotionally, he experiences deep regret and struggles with the fear of death. In his final moments, however, he attains a form of spiritual awakening, understanding that true meaning comes from compassion and authenticity rather than societal approval. The novella is a powerful and poignant exploration of life, death, and what it means to truly live. The ‘message’ of the novel, and Tolstoy’s philosophy on death, is that it is only in living our life for others that we overcome unhappiness and fear of death.

This may sound sanctimonious to some, but certainly transcendence of personal concerns seems important for people to find peace at the end-of-life. In a similar vein, the reflective essays of palliative care pioneer Dame Cicely Saunders provide insight into how death can be a journey toward gratitude and an invitation to hope. *Watch With Me: An Inspiration for Life In Hospice Care* is a collection of reflections, essays, and personal stories about the philosophy and practice of hospice and palliative care (Saunders and Saunders 2003). Saunders, a pioneer of the modern hospice movement, emphasizes the importance of providing compassionate, holistic care to terminally ill patients, addressing not just their physical pain but also their emotional, social, and spiritual needs. The book includes stories from patients, caregivers, and medical professionals, offering insight into the value of dignity, presence, and comfort in the face of death. It serves as both an inspiration and a guide for those involved in end-of-life care, reinforcing the idea that dying can be a meaningful and supported experience when approached with empathy and understanding.

Saunders also provides a succinct formulation of the challenge that physicians face in caring for patients at the end-of-life: *“We have to learn how to feel ‘with’ patients without feeling ‘like’ them if we are to give the kind of listening and steady support that they need to find their own way* through”. This is perhaps one of the biggest obstacles to genuine accompaniment of patients by physicians– the obstacle of learning how to *feel with* while not being able to *feel like* your patients.

Across these stories and narratives, a unifying theme is a reflection on the *good*,* true*,* and beautiful*. At its core, the medical humanities is a calling to see that which is “behind” the illness: the human person. It is a calling to even explore that which is “behind” the person, in the sense that, it is a call to reflect on the meaning of life (and therefore death), the ends of health, and the meaning of pain and suffering. And there is no more important moment in a person’s life than a person’s death, which is a culmination of all of the events, meaning, suffering, and joys in a person’s life. The medical humanities can provide a broader humanistic account of mortality, dying, and death, such that physicians can approach the care of patients with a more holistic understanding of the human person and not just a reductionistic approach to medical care.

## Discussion and recommendations

On the one hand, each person’s death is unique and everyone has their own individual personal biography. On the other hand, there is a collective wisdom contained within the medical humanities that can guide physicians in their approach to caring for patients at the end-of-life. We do well, then, to explore ways to make medical humanities a more central priority in medical education particularly with respect to death and dying.

It is important to highlight some promising recent contributions to medical humanities. The work by physicians using arts and visual-based media in medical training by Chisolm, Katz, Gaufberg, among others, have helped generations of trainees reflect on deeper themes on connection, death and dying, self-care, and humanistic care (Katz and Khoshbin 2014) (Chisolm et al. 2021) (Gaufberg et al. 2023). One example is the AAMC’s the Fundamental Role of Arts and Humanities in Medical Education (FRAHME) initiative which looks into the larger question of the importance of medical humanities in medical education in the 21st century (Howley et al. 2020). Another is the Neurohumanities Network, an effort to create a core curriculum exploring the humanities in neuroscience and neurology to augment neurology education at the intersection of the arts, humanities, and neurosciences (Doolittle et al. 2025). The Hippocratic Society is a physician-led initiative, growing chapters at academic medical institutions and chapters, providing a space for reflection on the bigger questions in medicine and the formation of physicians and clinician trainees (Hippocratic and Society 2024). There have also been initiatives at the patient level, such as the “My Life, My Story” initiative, which aims to enhance patient-centered care by integrating Veterans’ life stories and narratives into their clinical care, and the “I am Living” Australian public awareness campaign and website that seeks to expand understanding, and address community anxiety and some of the fears around the deterioration of health, dying, death, loss and grief (Walker et al. 2021).

It is also important to note that national contexts may differ, given the diversity in medical training programs. There are unique challenges arising from differences in varied socio-cultural contexts that should be taken into account. However, as evidenced by The Lancet Commission’s recent work bringing together experts to study the role of the humanities in the training of oncology residents on a global scale (Rodin et al. 2023), there is an increased global focus on the importance of integration of humanities to supplement formation of physicians in these questions that touch on the existential, anthropological, philosophical, and spiritual.

In an already crowded medical undergraduate curriculum, and in the intensity of schedules and debates over sustainability in resident schedules, the main question is, where, therefore, can the medical humanities be integrated into medical training? There have been many experts who have reflected on these questions before us, and therefore, we do not presume here to give definitive answers to challenging questions. However, here, we explore three themes for reflection on possible ways forward to humanize medicine, especially at the end-of-life: (1) physician discretionary space for identity formation and reflection, (2) a metaphysical approach to professionalism and ethics in the curricula, (3) attending physicians modeling character and virtue, and (4) a greater emphasis on patient narratives in clinical care.

### A physician discretionary space

The need for reflection around the care of a patient at the end-of-life revolves around both the physician’s confrontation that death does not have exceptions and that he/she will also experience death at some point, and the finality of death experienced through his/her patient. Studies consistently show that patient deaths are emotionally impactful, and without proper processing (Sansone and Sansone 2012), can lead to a sense of burnout among physicians (Redinbaugh et al. 2003). But that there is minimum time or space available for physicians to process patient death. Therefore, the physician is left only with his/her own practices, which, on a personal level, may or may not be developed.

This is where a physician’s discretionary space for processing is so vital in the processing of intensely emotional events. However, for physicians, who constantly experience patient suffering and death, the direct dwelling on experiences can be both challenging to directly confront. It is exactly in these spaces where the humanities, which can provide a more neutral medium, such as art, literature, music, or narrative, to process death and dying, and a reflective and intentional approach to one’s ongoing work in the hospital.

### Metaphysics in the formation of physicians

There is not currently a unified approach to formation in ethics and/or professionalism in medical training, and very little that is meta-physically based. The reasons for this are many, but one of the contributing factors may be the overall move of higher education away from an understanding of the university as a space for both intellectual and moral formation, to limiting its role to just intellectual formation. The university, one’s *alma mater*, or *nourishing mother*, was initially conceptualized as a place for the formation not only of the mind, but also the body and soul (Miller 2011). Though the current intellectual approach to higher education positively allows for exposure to a diverse array of ideas and topics, it comes at a loss of a deeper meta-physical approach of what it means to be human, what the meaning of suffering is, and what it means to die well, as these require a deeper moral, anthropological, and philosophical understanding and approach.

However, even if we do not perhaps have a unified approach to these deeper philosophical questions, to discard the exploration of these questions themselves leaves the work of medicine hollowed out, without a deeper ethos in which a trainee, who has just experienced his/her first patient death, can lean on to try to wrestle within oneself the meaning of life and death, and the meaning of suffering which he/she has committed him/herself to in this profession.

Therefore, explorations and reflections of the “why” behind what trainees are doing or experiencing is critical in the formation of early learners, whereas the current curricula focus on the “what” of ethics. However, as we have explored above, the main role of the humanities is an awakening of the deeper questions of the “why” as well as a reflection on the good, true, and aesthetic of the practice of medicine itself. Ultimately, death is a meta-physical question, though physicians interact with death on a very corporeal level. Jeff Bishop has written lucidly of how physician trainees are often first exposed to dead bodies in a cadaver lab before encountering living patients in a medical clinician (Bishop 2011). This somewhat confronting initial encounter with a dead body in clinical context can condition the way in which physicians go on to view not just living bodies throughout their career, namely, as an “anticipatory corpse”.

To counteract the onset of a metaphysics that frames the human body more as a machine moving through space rather than a living, breathing, organic system, we may need to introduce basic metaphysical concepts into medical training. One example might be an Aristotelian account of the soul whereby the human body is framed not as a machine but rather as an organic body possessed of a rational, animating soul. I would also be helpful to describe and emphasise the importance of character and virtue, and the understanding that virtues, which are habitual dispositions towards the *good*, allow physicians to more fully realise the aims of medicine and the goals of care.

### Patient narratives in clinical care

Medical students are taught early on the importance of taking a social history. However, the format in which the social history takes place is often limited to a biomedicine adjacent approach: whether the patient is taking alcohol, tobacco, or other drugs, or whether the patient is engaging in other activities that may have a direct impact on the biological model of health. However, the true social history of the patient, the patient’s narrative and story, are often missed, despite the fact that they are so integral to patient care. Robert Coles, a physician, writer, and teacher at Harvard University, stressed the importance of patient narratives, in the formation of physicians’ moral and professional development (Coles et al., 2012). He stressed how narratives help doctors cultivate empathy, ethical sensitivity, and a deeper understanding beyond the biological. French philosopher Paul Ricouer also reflected on the importance of narrative in a manner that is relevant for clinicians (Flanagan 2019). Specifically, his work helps show how patient narratives (Karkabi et al. 2014) reconfigure not only the identity of the patient as he/she experiences the illness, but also the clinician, whose own identity is also reconfigured through the reception of that patient story (Russo 2021).

Especially at the end-of-life, conversations around goals-of-care are couched in what is most important to the patient, a patient’s hopes and dreams as well as the patient’s worries and fears. In the last chapters of a patient’s life, treatment paradigms do not ever fit well into a particular patient’s case, because the individuality of the patient’s meaningfully lived life is what determines the last phases of medical treatment.

In that way, how a physician incorporates in the “social history” of the patient, the patient’s narrative, is crucial even in the formulation of the plan for the patient, and is ever more crucial at the end-of-life. An oncologist who is able to integrate a treatment plan which takes into account a patient’s desire to spend as little time in the hospital as possible, will require a certain degree of creativity with infusions, labs, and follow-ups than may be typically required. It requires a humanizing of the care, because the care then becomes about this particular person in front of me as a clinician.

There are various ways to bring narrative into the patient encounter, including starting off with a new consultation by asking about the person and who he/she is and what is most important to them, as one also comes to understand their disease process. It requires a certain frameshift, where the diagnosis and treatment is important, but that it is seen within the context of the person’s journey and story, where he/she comes from, and where he/she is going.

## Conclusion

In this essay we have explored an enduring challenge for medical education, namely, providing a humanistic education for medical students who are preparing to care for patients at the end-of-life. We have sought to position the medical humanities as one potential means of addressing the inadequate preparation that doctors receive in this area. We are not pretending that the medical humanities are a miracle solution to the manifold challenges that doctors might encounter in end-of-life contexts. But we do think that a deep humanistic formation– focused on making explicit key metaphysical assumptions that doctors might deploy in their practice as well as bringing doctors more into contact with patient narratives surrounding death– will help to ensure that patients in our health systems receive an excellent, holistic care from doctors and other clinical staff as they prepare for death. Meaningful change is needed in the curricula that underpin clinical education in medical schools, but given the gravity of the problem, we hope that medical educators will be willing to give this task due priority as they seek to redesign their courses and clinical training programs for students.

## Data Availability

No datasets were generated or analysed during the current study.

## References

[CR1] A Definition of Irreversible Coma. Report of the ad hoc committee of the Harvard medical school to examine the definition of brain death, 1968. *Journal of the American Medical Association* 205, 337–340. 10.1001/jama.1968.03140320031009 5694976

[CR2] Baker, M., J. Wrubel, and M. W. Rabow. 2011. Professional development and the informal curriculum in end-of-life care. *Journal of Cancer Education* 26:444–450. 10.1007/s13187-011-0199-x 21350931 PMC3161185

[CR3] Bishop, J. P. 2011. *The anticipatory corpse: Medicine, power, and the care of the dying*. University of Notre Dame.

[CR4] Bleakley, A. 2019. Invoking the medical humanities to develop a #medicinewecantrust. *Academic Medicine* 94:1422–1424. 10.1097/ACM.0000000000002870 31299677

[CR5] Brown, S. 2019. Why many Doctors still find it difficult to talk about dying with patients. *Cmaj* 191:E22–E23. 10.1503/cmaj.109-5691 30617232 PMC6312518

[CR6] Cassel, E. J. 1982. The nature of suffering and the goals of medicine. *New England Journal of Medicine* 306:639–645. 10.1056/NEJM198203183061104 7057823

[CR7] Chisolm, M. S., M. Kelly-Hedrick, and S. M. Wright. 2021. How Visual Arts–Based Education Can Promote Clinical Excellence. Academic Medicine 96. 10.1097/ACM.0000000000003862 33264111

[CR8] Coles, R., R. Testa, W. Berry, R. Carver, A. Chekhov, A. Fadiman, K. R. Jamison, A. Lorde, L. Moore, and R. N. Remen et al. 2012*. A life in medicine: A literary anthology*. The New Press.

[CR9] Delbani, R., C. J. Barnes, and M. Shamy. 2024. The humanities in palliative medicine training: Perspectives of academic palliative medicine physicians and trainees. *BMC Medical Education* 24:1337. 10.1186/s12909-024-06295-0 39567976 PMC11580182

[CR10] Doolittle, J., C. Lewis, G. Gheihman, M. Rosso, C. Palmer, and M. Stanley. 2025. Launching the neurohumanities network: An online, Trainee-Led, transdisciplinary collaborative effort to foster Understanding of the arts, humanities, and neurosciences (P5-5.004). *Neurology* 104:3886. 10.1212/WNL.0000000000211302

[CR11] Flanagan, T. 2019. *Narrative medicine in hospice care: Identity, practice, and ethics through the Lens of Paul Ricoeur, studies in the thought of Paul Ricoeur*. Lexington Books.

[CR12] Fox, D. M. 1985. Who we are: The political origins of the medical humanities. *Theoretical Medicine* 6:327–341. 10.1007/BF00489733 3904071

[CR13] Gaufberg, E., DiGiovanni, Evans,Brooke, Rutberg, Pooja, and M. S. Chisolm. 2023. What’s art got to do with it?—Transfer of learning in museum-based health professions education. International Review of Psychiatry 35, 672–681. 10.1080/09540261.2023.2288299 38461382

[CR14] Gawande, A. 2014. *Being mortal: Medicine and what matters in the end., being mortal: Medicine and what matters in the end*. New York, NY, US: Metropolitan Books/Henry Holt and Company.

[CR15] Hafferty, F. W., E. H. Gaufberg, and J. F. O’Donnell. 2015. The role of the hidden curriculum in on doctoring courses. *AMA J Ethics* 17:130–139. 10.1001/virtualmentor.2015.17.02.medu1-1502 25676226

[CR16] Hippocratic, and Society. 2024. The Hippocratic Society. URL https://hippsoc.org/.

[CR17] Howley, L., E. Gaufberg, B. King, and A. Colleges. 2020. of A.M., The Fundamental Role of the Arts and Humanities in Medical Education. Association of American Medical Colleges.

[CR18] Kalanithi, P., A. Verghese, and L. Kalanithi. 2016. *When breath becomes air / Paul Kalanithi; foreword by Abraham Verghese*. New York: Random House.

[CR19] Karkabi, K., H. S. Wald, and O. Cohen Castel. 2014. The use of abstract paintings and narratives to foster reflective capacity in medical educators: A multinational faculty development workshop. *The Journal of Medical Humanities* 40:44. 10.1136/medhum-2013-010378 PMC4033026 24273319

[CR20] Katz, J. T., and S. Khoshbin. 2014. Can visual arts training improve physician performance? Trans Am Clin Climatol Assoc 125, 331–41; discussion 341–342. PMC4112699 25125749

[CR21] Miller, E. J. 2011. John Henry Newman’s Idea of Alma mater. *Newman Studies Journal* 8:19–28. 10.1353/nsj.2011.0017

[CR22] Redinbaugh, E. M., A. M. Sullivan, S. D. Block, N. M. Gadmer, M. Lakoma, A. M. Mitchell, D. Seltzer, J. Wolford, and R. M. Arnold. 2003. Doctors’ emotional reactions to recent death of a patient: Cross sectional study of hospital Doctors. *Bmj* 327:185. 10.1136/bmj.327.7408.185 12881257 PMC166122

[CR23] Rodin, G., M. Skelton, N. Bhoo-Pathy, O. Dewachi, M. Li, D. Trapani, E. Smyth, M. Banegas, N. Salins, K. Unger-Saldaña, C. Zimmermann, and R. Sullivan. 2023. Establishing a lancet oncology commission on the humanitarian crisis of cancer. *The Lancet Oncology* 24:835–837. 10.1016/S1470-2045(23)00346-7 37541269

[CR24] Russo, M. T. 2021. Ricoeur’s hermeneutic Arc and the narrative turn in the ethics of care. *Medicine, Health Care and Philosophy* 24:443–452. 10.1007/s11019-021-10020-9 33914221 PMC8349331

[CR25] Sansone, R. A., and L. A. Sansone. 2012. Physician grief with patient death. *Innov Clin Neurosci* 9:22–26. PMC3366454 22666638

[CR26] Saunders, C. M., and C. Saunders. 2003. *Watch with me: Inspiration for a life in hospice care*. Observatory.

[CR27] Sullivan, A. M., M. D. Lakoma, and S. D. Block. 2003. The status of medical education in end-of-life care: A National report. *Journal of General Internal Medicine* 18:685–695. 10.1046/j.1525-1497.2003.21215.x 12950476 PMC1494921

[CR28] Sutherland, R. 2019. Dying Well-Informed: The need for better clinical education surrounding facilitating End-of-Life conversations. *The Yale Journal of Biology and Medicine* 92:757–764. 31866792 PMC6913833

[CR29] Tolstoy, L. 1973. graf, 1828–1910, The death of Ivan Ilych. New York: Health Sciences Pub. Corp., 1973.

[CR30] Van Aalst-Cohen, E. S., R. Riggs, and I. R. Byock. 2008. Palliative care in medical school curricula: A survey of united States medical schools. *Journal of Palliative Medicine* 11:1200–1202. 10.1089/jpm.2008.0118 19021481

[CR31] Viney, W., F. Callard, and A. Woods. 2015. Critical medical humanities: Embracing entanglement, taking risks. *Medical Humanities* 41:2–7. 10.1136/medhum-2015-010692 26052111 PMC4484495

[CR32] Walker, E., E. Bruns, and G. Dhaliwal. 2021. The VA my life my story project: Keeping medical students and veterans socially connected while physically distanced. *Fed Pract* 38:568–573. 10.12788/fp.0208 35177886 PMC8843003

